# Corrosion Induced on Aluminum by Biodiesel Components in Non-Oxygen Environments

**DOI:** 10.3390/ma17081821

**Published:** 2024-04-16

**Authors:** Fabiola Vergara-Juarez, Jesus Porcayo-Calderon, Juan Pablo Perez-Orozco, Macdiel Emilio Acevedo-Quiroz, Victoria Bustos-Terrones, Alfredo Quinto-Hernandez

**Affiliations:** 1 Tecnológico Nacional de México/Instituto Tecnológico de Zacatepec, Departamento de Ingeniería Química y Bioquímica, Calzada Tecnológico 27, Zacatepec de Hidalgo 62780, Morelos, Mexico; fabizvj@gmail.com (F.V.-J.); juan.po@zacatepec.tecnm.mx (J.P.P.-O.); 2 Departamento de Ingeniería Química y Metalurgia, Universidad de Sonora, Hermosillo 83000, Sonora, Mexico; jesus.porcayo@unison.mx; 3 Tecnológico Nacional de México/Instituto Tecnológico de Zacatepec, Departamento de Ciencias Básicas, Calzada Tecnológico 27, Zacatepec de Hidalgo 62780, Morelos, Mexico; macdiel.aq@zacatepec.tecnm.mx; 4 Dirección Académica de Ingeniería en Tecnología Ambiental, Universidad Politécnica del Estado de Morelos, Boulevard Cuauhnáhuac No. 566, Col. Lomas del Texcal, Jiutepec 62550, Morelos, Mexico; vbustos@upemor.edu.mx

**Keywords:** biodiesel, fatty acid methyl esters, corrosion, unsaturation, C=C bond, loss mass, polarization potentiodynamic curves, linear polarization resistance, electrochemical impedance spectroscopy

## Abstract

Biodiesel is a mixture of saturated and unsaturated Fatty Acid Methyl Esters (FAMEs) whose composition affects the corrosion behavior of metal containers during storage. This study examines the effect of the C=C bond present in selected FAMEs (Methyl Stearate, Methyl Oleate, and Methyl Linoleate) in aluminum corrosion in the absence of oxygen. First, mass loss assays were carried out at 100, 200, and 280 °C for 1000 h using pure Methyl Stearate (MS), 5% Methyl Oleate in Methyl Stearate (MS-5% MO), and 5% Methyl Linoleate in Methyl Stearate (MS-5% ML). Next, chemical changes in FAMEs were studied using FTIR, TGA, and GC/MS. SEM/EDS analysis allowed us to inspect the aluminum surfaces and their chemical characterization. We estimated higher corrosion rates for MS assays than those of unsaturated methyl ester mixtures. In a separate set of experiments, we used electrochemical techniques (potentiodynamic polarization, linear polarization resistance, and electrochemical impedance spectroscopy) to investigate aluminum corrosion induced by thermal-degraded products from FAMEs at 100, 200, and 280 °C for 300 h able to dissolve in aqueous extracts. These electrochemical experiments revealed that the products in the aqueous extracts from the unsaturated methyl ester mixture form a passive layer on the Al surface thicker than pure MS at the corresponding degradation temperatures.

## 1. Introduction

Fossil fuel depletion and its environmental impact have triggered our interest in obtaining alternative energy sources. Biodiesel is suitable for replacing conventional fuels, given its renewable and non-toxic nature, inherent lubrication, and very low sulfur content [1]. Biodiesel is a mixture of Fatty Acid Methyl Esters (FAMEs) and Fatty Acid Ethyl Esters (FAEEs) whose molecular structures vary in their number of carbon atoms, presence of C=C bonds in the alkyl chain, and composition at about ~10% oxygen and ~40% unsaturated esters [2,3]. Although the massive use of biodiesel may become a reality, several factors prevent its implementation, including its corrosive nature and relatively easy degradation at moderate temperatures [4,5]. The presence of unsaturated C=C bonds and oxygen in the components of biodiesel, besides moisture absorption and catalyst impurities resulting from biodiesel synthesis, could be the leading causes that induce biodiesel degradation and metal deterioration [6].

Unsaturated C=C bonds in biodiesel components are a factor that makes it more susceptible to oxidation than diesel [7,8]. A plausible explanation is that the electron exchange reaction usually starts in an allylic position to the C=C double bond [9,10,11]. Even more, experimental work suggests polyunsaturated methyl esters are more vulnerable to thermal degradation than monounsaturated or saturated ones [12]. Therefore, biodiesel with a high content of unsaturated esters could be more corrosive. Understanding the effect of unsaturated methyl esters is crucial to predicting biodiesel’s performance at different operation temperatures and in the corrosive impact on metal surfaces once in contact. Thermal degradation, exposure time, and biodiesel oxidation also induce corrosion on metal surfaces, as shown for copper, brass, bronze, aluminum, cast iron, and carbon steel [11,13,14]. In general, copper and copper-based alloys are significantly more prone to corrosion due to pitting, according to mass loss assays [15,16,17,18,19], electrochemical impedance spectroscopy experiments (EIS) [20,21], and morphological characterization (SEM/EDS) [18]. Also, the metals and alloys mentioned above generally show increased corrosion in response to higher temperatures and exposure time in biodiesel [16,17,22,23]. The degree of thermal degradation of methyl esters is directly related to biodiesel’s performance [19] and remains associated with biodiesel oxidation capacity. During this oxidation, peroxides and hydroxyperoxides are formed [24] and converted later into aldehydes, ketones, and volatilized acids. All these molecules have potential corrosive characteristics.

Investigations on aluminum corrosion induced by biodiesel are available. Up to now, the main focus reported is on corrosion rates obtained via mass loss assays using biodiesel synthesized from different oil feedstocks. Such assays conclude that the corrosion phenomena are relevant only at longer exposure times [15,16,18,25]. The detection of corrosion products on aluminum surfaces has also been possible using electrochemical techniques (EIS, open circuit, and anodic polarization measurements) and is associated with decreased aluminum surface activity [26]. Several recent reviews describing the corrosion behavior of biodiesel can be found elsewhere for aluminum, copper, and several other metals [27,28,29]. Nevertheless, studies explaining corrosion mechanisms from individual saturated or unsaturated biodiesel components on metal surfaces remain undeveloped.

The most significant concern in the commercial use of biodiesel in the automotive sector is the degradation or failure of the metal components that constitute the engine, such as aluminum, copper, and stainless steel [30]. Identifying corrosion mechanisms in these metals is crucial to introducing effective solutions. Thus, this work reports the corrosive impact on aluminum within a non-oxygen environment caused by methyl esters and their thermal degradation products using mass loss and electrochemical (potentiodynamic polarization curves, linear polarization resistance, and electrochemical impedance spectroscopy) measurements. We used non-oxygen environments to exclusively identify the effect of the C=C bond and temperature on the corrosion behavior of the methyl esters on aluminum. Methyl esters considered in this work are the saturated biodiesel component Methyl Stearate (MS, 18:0) and their unsaturated analogs, Methyl Oleate (MO, 18:1) and Methyl Linoleate (ML, 18:2).

## 2. Materials and Methods

### 2.1. Materials

The analytical-grade methyl esters (Methyl Stearate, Methyl Oleate, and Methyl Linoleate) used in this study were purchased from Sigma-Aldrich (St. Louis, MO, USA) without further purification. Aluminum coupons (>99.99%) were used as test specimens or working electrodes.

### 2.2. Mass Loss Assays

Mass loss assays on aluminum coupons (dimensions: 18 × 24 × 3 mm) were carried out by hanging them in a corrosive medium. A 2 mm diameter hole was drilled near the edge where each coupon was suspended for immersion. The metal samples were polished with silicon carbide abrasive paper (240, 600, and 1000), washed with distilled water, and finally with acetone. In each assay, three coupons were used. The corrosive media were pure Methyl Stearate (MS) and mixtures of 5% Methyl Oleate in Methyl Stearate (MS-5% MO) and 5% Methyl Linoleate in Methyl Stearate (MS-5% ML). The assays were performed at 100, 200, and 280 °C for 1000 h of immersion in a non-oxygen environment created by bubbling the mixture with nitrogen. After immersion, corrosion rates were determined using the procedure established in the ASTM G31-21 standard [31], according to Equation (1).

(1) $$Corrosion\;rate\;(mpy)=\frac{K\times W}{A\times T\times D}$$

where K is a corrosion constant (3.45 × 10^6^), T is the exposure time in hours, A is the exposure area in cm^2^, W is the mass loss in g, and D is the metal density (2.71 g/cm^3^).

### 2.3. Surface Characterization

The surface morphology of the aluminum specimens after immersion into corrosive media (MS, MS-5% MO, and MS-5% ML) was examined with scanning electron microscopy (SEM). We used a Hitachi S5500 instrument at 10 kV, which provided a nanometric resolution and allowed us to identify the chemical composition of the film formed on the specimen’s surface using a coupled Energy-Dispersive X-ray (EDS) Spectrometer (Hitachi, Tokyo, Japan).

### 2.4. Characterization of Chemical Modifications

We used Fourier-Transform Infrared (FTIR) spectroscopy and thermogravimetric analysis (TGA) to monitor the evolution of thermal degradation in the methyl ester mixtures, as indicated in the mass loss assays. For FTIR measurements, an FTIR-ATR Perkin Elmer Spectrum One spectrometer (PerkinElmer, Shelton, CT, USA) was employed at a resolution of 4 cm^−1^ during 16 scans, in the range of 4000–500 cm^−1^. We worked with a Mettler Toledo TGA 2 Star System analyzer (Mettler-Toledo, Columbus, OH, USA) for TGA using a scanning rate of 10 °C/min. Samples of ~10 mg were heated at 30–700 °C in a nitrogen atmosphere. During TGA experiments, the flow rate employed was 30 mL min^−1^. After thermal degradation, each sample mixture’s FAME (Fatty Acid Methyl Ester) profile was evaluated using gas chromatography coupled with mass spectrometry (GC/MS). The analysis was performed on an Agilent Technology 6890 gas chromatograph (Agilent Technologies, Santa Clara, CA, USA) interfaced with a 5973 N mass spectrometer. This system was equipped with an HP-5MS capillary nonpolar column (30 m, ID: 0.25 mm, film thickness: 0.25 μm) and connected to an ion trap detector operating in electron impact mode at 70 eV. Helium was used as the carrier gas at a 1 mL min^−1^ flow rate, and the injection volume was 20 μL (in HPLC grade CHCl_3_). The oven temperature was programmed to increase from 50 °C to 230 °C at 2 °C/min. The results were compared using the NIST/EPA/NIH Mass Spectral Library, version 1.7a/ChemStation.

### 2.5. Electrochemical Assays

Electrochemical measurements were performed in a conventional three-electrode cell using a Gamry Potentiostat/Galvanostat/ZRA (Interface 1010E) (Gamry Instruments, Warminster, PA, USA). Platinum wires were used as the reference and counter electrodes. Previous work has reported using platinum as a reference electrode in electrochemical measurements and shown that using platinum as a reference electrode instead of conventional electrodes does not alter the information’s content [32,33,34,35]. The working electrode was an aluminum-made plate (reaction area ~1 cm^2^), to which a copper conductor wire was welded on one of its faces, followed by encapsulation in epoxy resin. Before each test, we ensured the electrodes’ working surface was polished with abrasive paper. The corrosive medium used was the aqueous extract of the thermal degradation products for the different methyl ester mixtures (MS, MS-5% MO, and MS-5% ML). Each mixture was degraded in a hydrothermal reactor for 300 h at 100, 200, and 280 °C. Before degradation, a non-oxygen environment was also created by bubbling each mixture with nitrogen gas for 1 h. Subsequently, the reactor was hermetically closed. After the thermal degradation, we obtained an aqueous extract by vigorously mixing the thermally degraded methyl esters and distilled water with a 1:1 volume ratio for one hour. Then, we kept a slow mixture for another 24 h.

Electrochemical tests consisted of potentiodynamic polarization curves, linear polarization resistance, and electrochemical impedance spectroscopy measurements. For polarization measurements, the oxygen in the electrochemical cell was displaced by bubbling nitrogen gas for 10 min. Potentiodynamic polarization curves were carried out by polarizing the working electrode from −500 mV to 1700 mV concerning its corrosion potential at a scanning speed of 1 mV/s. Extrapolating the Tafel regions allowed us to estimate electrochemical parameters (E_corr_, I_corr_, anodic, and cathodic slope). Linear polarization measurements were obtained at one-hour intervals by polarizing the electrode ±15 mV concerning its potential at a 10 mV/min scan rate. Electrochemical impedance spectroscopy measurements were carried out by applying a perturbation of 20 mV alternating current in the frequency range of 100 kHz to 0.01 Hz. Each test lasted 100 h, using steps of 25 h at room temperature.

Distilled water is characterized by high resistivity. An arrangement was made on the electrochemical cell electrodes to verify the previously reported ohmic drop for a system with high resistivity like this medium [36]. The separation distance between the electrodes is a parameter that contributes to the measurement of electrochemical assays, favoring the acquisition and accuracy of the results in media with high resistivity [37]. Considering previous works [38,39], in order to ensure that the resistance is directly proportional to the separation between the electrodes, the platinum wires were placed on the working electrode, covering the complete surface of the sample, so that the separation between them was 2 mm. Due to the solution’s high resistivity, automatic IR compensation was applied in potentiodynamic tests.

## 3. Results and Discussion

### 3.1. Mass Loss Measurements

**Figure 1:**
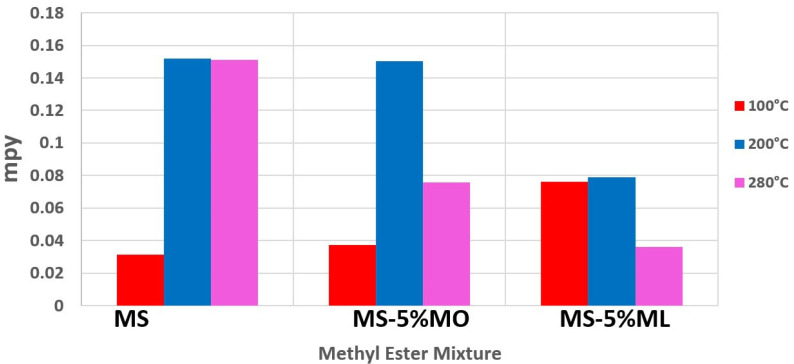
Corrosion rates obtained using mass loss assays for aluminum samples after 1000 h of immersion at 100 °C (red bar), 200 °C (blue bar), and 280 °C of Methyl Stearate (MS), 5% Methyl Oleate in Methyl Stearate (MS-5% MO), and 5% Methyl Linoleate in Methyl Stearate (MS-5% ML) mixtures.

Figure 1 shows the corrosion rates calculated for the mass loss of aluminum samples exposed to the different methyl ester mixtures in mpy. We estimated low corrosion rates (0.30 to 7.3 × 10^−2^ mpy) for all methyl ester mixtures when the thermogravimetric assays were carried out at 100 °C. Our thermal degradation assays’ highest corrosion rates (15.3 × 10^−2^ mpy) correspond to the MS samples at 200 and 280 °C. At 200 °C, the corrosion rate for MS-5% MO is similar to that of MS. However, we found a decrease in the corrosion rates for MS-5%MO at 280 °C (7.5 × 10^−2^ mpy), MS-5% ML at 200 °C (7.5 × 10^−2^ mpy), and 280 °C (3.5 × 10^−2^ mpy). Corrosive behavior in the samples at 100 °C is attributed to the high thermal stability of MS below 190 °C, as shown later with TGA analysis. CG/MS corroborated this behavior since no new compounds were detected in methyl ester samples treated at 100 °C. High corrosion rates in MS at 200 and 280 °C may be explained by the slow production of corrosive substances that gradually pass into the gaseous phase. This transfer could have provided enough time to interact with the metal surface. In contrast, since unsaturated esters with one or two unsaturations (MO, ML) had more fragile thermal stability, corrosive compounds obtained at 200–280 °C potentially were lost faster into the gas stream. No comparative values for these corrosion rates from pure MS, MO, or ML are available, but the effect of the unsaturation C=C on the corrosion rate in the mixtures is evident. Mass loss tests of biodiesel synthesized from several oils in the presence of oxygen have reported a similar tendency in corrosion rates [7,40,41]. Easily oxidizable biodiesel has many unsaturated molecules and is less corrosive. The results obtained by mass loss under oxidizable conditions corroborate that unsaturated molecules in biodiesel also decrease the corrosion rate on metal surfaces.

### 3.2. Surface Morphology

**Figure 2:**
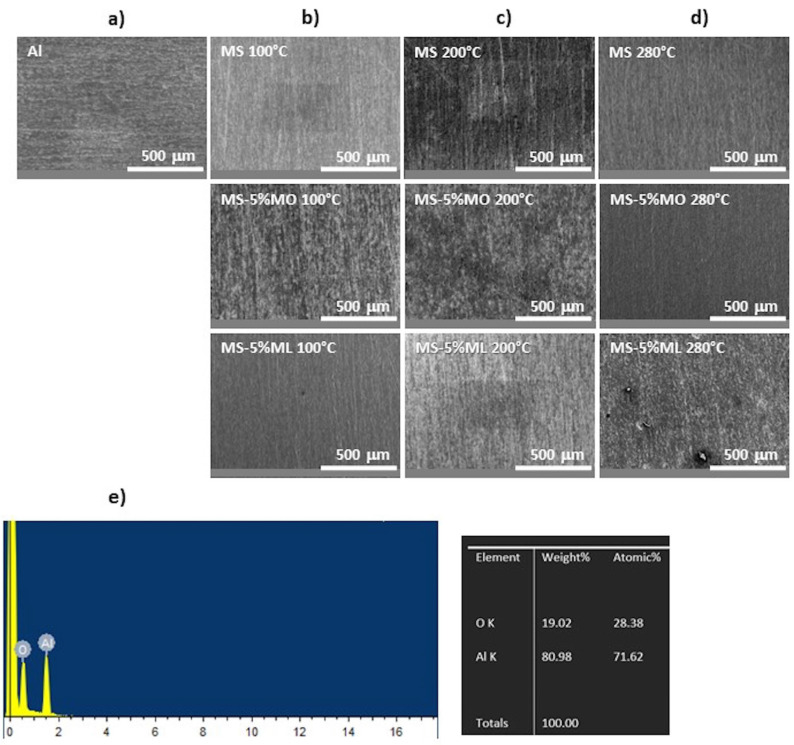
SEM microphotographs of aluminum specimens (**a**) before and after 1000 h of immersion in corrosive media (Methyl Stearate (MS), 5% Methyl Oleate in Methyl Stearate (MS-5% MO), and 5% Methyl Linoleate in Methyl Stearate (MS-5% ML)) at (**b**) 100 °C, (**c**) 200 °C, and (**d**) 280 °C. (**e**) Typical EDS spectrum of aluminum surfaces exposed to corrosive media.

Figure 2 shows the surface morphology using SEM of the aluminum specimens studied in this work. Before mass loss assays, we obtained a microphotograph of the original polished aluminum (Figure 2a). The entire surface is smooth and homogeneous. A similar morphology was observed for the aluminum surfaces exposed to the different corrosive media (Methyl Stearate (MS), 5% Methyl Oleate in Methyl Stearate (MS-5% MO), and 5% Methyl Linoleate in Methyl Stearate (MS-5% ML)) at 100, 200, and 280 °C (Figure 2b–d). However, typical EDS analysis for the aluminum surfaces resulting from the mass loss assays (Figure 2e) reveals the presence of Al_2_O_3_, not detected in the original polished aluminum. Al_2_O_3_ is a protective oxide responsible for the high corrosion resistance at all temperatures. The magnification of all images is 100x.

### 3.3. Characterization of Chemical Changes

**Figure 3:**
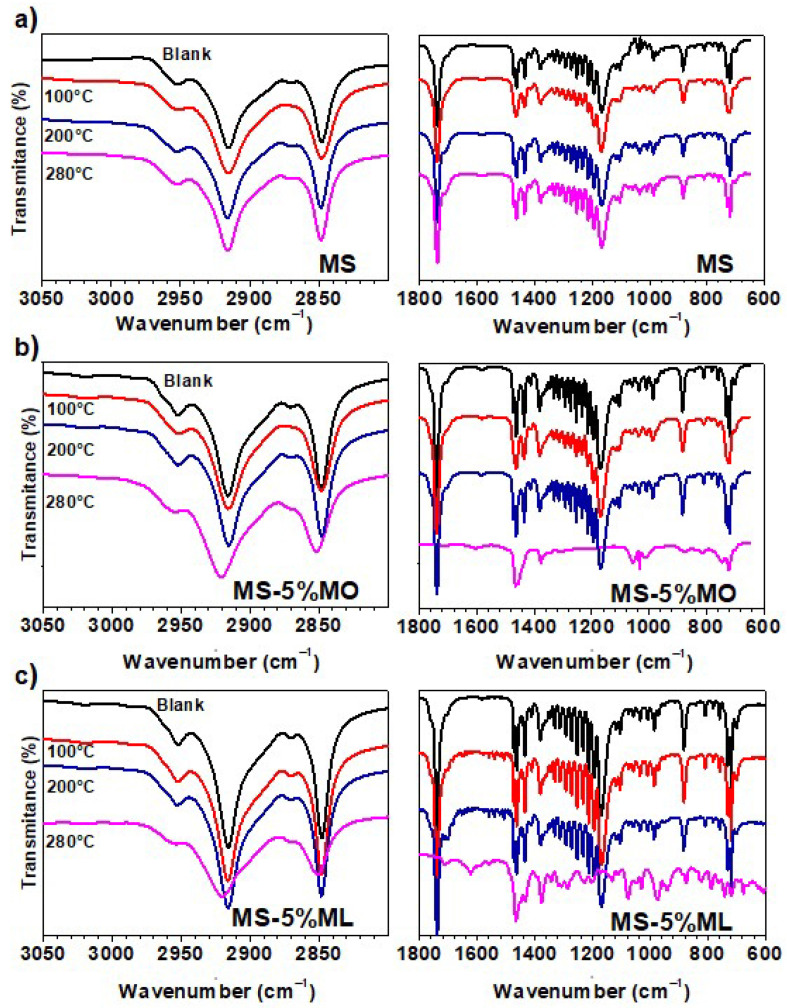
Fourier-Transform Infrared Spectroscopy of corrosive immersion media after 1000 h of the exposed metal samples. Corrosive immersion media were sampled at 100 °C (red line), 200 °C (blue line), 280 °C (pink line), and their blanks, and include (**a**) Methyl Stearate (MS), (**b**) 5% Methyl Oleate in Methyl Stearate (MS-5% MO), and (**c**) 5% Methyl Linoleate in Methyl Stearate (MS-5% ML) mixtures.

Fourier-Transform Infrared (FTIR) analysis identified the chemical species produced during the thermal degradation assays. Figure 3 shows the FTIR spectra recorded for samples of (a) MS, (b) MS-5% MO, and (c) MS-5% ML mixtures degraded at 100, 200, and 280 °C after 1000 h of immersion, also including when the immersion tests began (blank). For clarity, the FTIR spectra are shown in the regions 3050–2800 cm^−1^ and 1800–600 cm^−1^, where the most essential absorption bands are located. The FTIR spectra of methyl ester mixtures sampled initially at the immersion tests are similar. They show characteristic absorption bands for C=O, C-O, and O-CH_3_ groups at about 1738, 1166, and 1379 cm^−1^, respectively. For the MS-5% MO and MS-5% ML mixtures, a small peak is visible at 3018 cm^−1^, indicating the presence of C=C [42]. All the ester mixtures under thermal degradation at 100 and 200 °C show unchanged characteristic bands, suggesting that chemical transformations are absent and that the evaporation of the samples mainly dominated the thermal process. At 280 °C, the absorption bands for the FTIR spectrum of MS are also unchanged; however, those FTIR spectra for the MS-5% MO and MS-5% ML mixtures indicate the production of new species. Here, the intensity of characteristic signals associated with C=O and C-O decreased, suggesting a degradation of the samples. Also, a band is present at 3018 cm^−1^ in the MS-5% ML but disappears in the MS-5% MO mixture. This signal may be due to moisture in MS-5% ML. This band implies that this mixture degrades and absorbs moisture faster than the others at 280 °C.

**Figure 4:**
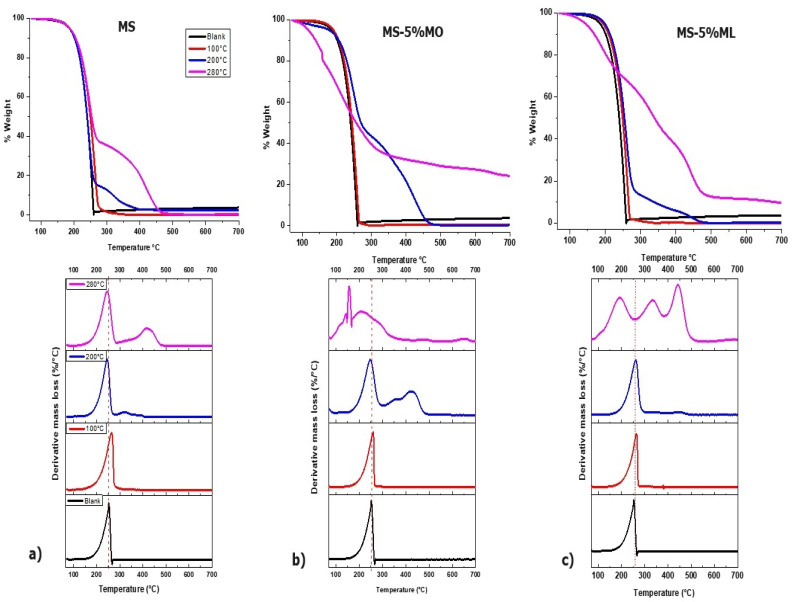
Thermogravimetric analysis (TGA) for the corrosive immersion media sampled at 100 °C (red line), 200 °C (blue line), 280 °C (pink line), and their blanks, after 1000 h of immersion. Corrosive immersion media were (**a**) Methyl Stearate (MS), (**b**) 5% Methyl Oleate in Methyl Stearate (MS-5% MO), and (**c**) 5% Methyl Linoleate in Methyl Stearate (MS-5% ML) mixtures. Lower panels correspond to the thermogravimetric derivative (TGD) curves of the corresponding TGA plots.

Figure 4 shows the TGA plots (top panels) for (a) MS, (b) MS-5% MO, and (c) MS-5% ML mixtures after 1000 h of immersion. The TGA plots were recorded for immersions sampled at 100 °C (red line), 200 °C (blue line), 280 °C (pink line), and a blank of MS (without thermal degradation). We also present the thermogravimetric derivative (TGD) curves of the corresponding TGA plots to identify the thermal stability and decomposition temperatures for the species produced during the degradation. Thermal behavior for all mixtures degraded to 100 °C proceeded through a single step and is similar to the blank of MS. Therefore, no chemical species were released into the gas stream at this temperature, and the mixtures volatilized only up to 200 or 280 °C. The thermal process for samples thermally degraded at 200 °C involves two steps, attributed to decomposition and volatilization. The two processes can be verified in the TGD curves at 200 °C. First, a mass loss of 80% is observed in the temperature range of 180–290 °C for MS and MS-5% ML, while for MS-5% MO, the mass loss is 50%. The second step shows that the mixtures containing unsaturated methyl esters are more thermally stable than the degraded MS. A mass loss of 90% occurred in 280–370, 300–480, and 290–490 °C for the MS, MS-5% MO, and MS-5% ML mixtures, respectively. These results are consistent with castor oil biodiesel thermal degradation reports at 210 °C, also characterized by a two-step process [43]. The newly formed high molecular weight compounds decompose at higher temperatures during the second step, given that the maximum degradation temperature increases with the number of C=C bonds in the mixtures. This statement is supported by the GC/MS analysis, where compounds with longer carbon chains compared to initial compounds are observed after the ester is degraded thermally.

TGA plots also reveal that all the methyl ester mixtures degraded at 280 °C remain thermally stable up to 120 °C. Subsequently, a constant mass loss occurs with a different tendency for each mixture. For pure degraded MS, only two processes occurred at 150–290 and 360–470 °C. In contrast, the thermal degradation of the MS-5% MO and MS-5% ML mixtures involves three steps attributed to decomposition and volatilization. The temperature ranges for the MS-5% MO are 100–180, 180–250, and 250–350°, and for the MS-5% ML are 100–250, 250–400, and 400–500 °C. Methyl esters degraded at high temperatures generate polymeric compounds [44]. Also, previous work [45] has reported that the decomposition of methyl esters leads to hydroperoxides, aldehydes, esters, ketones, acids, alcohols, and polymeric species. In addition, high temperatures significantly affect methyl esters by yielding polymerized compounds and increasing weight, for instance, in biodiesel [44,46].

Suppose the methyl esters contain C=C bonds in the alkyl chain; in that case, this unsaturation causes inter- and intramolecular cross-linking, making the polymerization more efficient [24] and giving rise to oligomers with a higher degradation temperature. Our TGA study focused on the compounds generated in MS, MS-5% MO, and MS-5% ML at 280 °C corroborates the effect that the presence of C=C bonds has on the production of non-oxidative compounds by thermal degradation, observing that the presence of unsaturations in the esters favors the production of oligomers with higher degradation temperatures. In all cases, the increase in residual mass was directly proportional to the rise in aging temperature, as reported previously [47], where forming compounds with higher molecular weight in biodiesel changes the thermogravimetric behavior.

The formation of chemical species during the thermal degradation of the methyl ester mixtures was investigated via GC/MS. All ester mixtures under thermal assays at 100 °C were identified exclusively by the same components of their corresponding blanks, thus confirming the results obtained via FTIR and TGA. Table 1 lists the main products obtained from the MS, MS-5% MO, and MS-5% ML mixtures at the other degradation temperatures (200 and 280 °C). Products formed through thermal degradation of MS at 200 °C are the methyl esters listed in Table 1 as Product 1 (Eicosanoic acid, methyl ester) to Product 3 (Hexadecanoic acid, methyl ester). Some Octadecanoic acid, methyl ester, or Methyl Stearate remained unreacted. By increasing the aging temperature (280 °C), n-heptane (Product 6) was detected in addition to other esters (Heptadecanoic acid, methyl ester and Decanedioic acid, dibutyl ester). For methyl ester mixtures containing C=C bonds (MS-5% MO and MS-5% ML), Methyl Oleate (MO) and Methyl Linoleate (ML) were unobserved at either 200 or 280 °C. The resulting products of the thermal assay at 200 °C in MS were also found in the mixtures for both MS-5% MO and MS-5% ML; therefore, these components may be unrelated to MO or ML. At 200 °C, MS-5% MO produced Nonadecanoic acid, methyl ester (Product 7), and a higher molecular weight compound (Octadecanoic acid, octyl ester, Product 8). For MS-5% ML, n-Heptadecane (Product 6) and Bis(2-ethylhexyl)phthalate (Product 16) were also detected. Unsaturated carbonated chains remained unobserved for samples degraded at 280 °C. This thermal condition allowed for the production of large alkanes (Products 9 to 14) and an oxo ester (Products 15) after aging the MS-5% MO mixture in the inert atmosphere. In contrast, shorter alkanes (Products 6, 7, 14, and 18) were observed in the mixture MS-5% ML. This result differs from previous works [48,49] that report n-alkanes, n-alkenes, methyl ketone, alcohols, aldehydes, and carboxylic acids as species formed from MS, MO, or ML in the presence of oxygen. Since our experiments were carried out without oxygen, the products in Table 1 were only produced from thermal degradation.

### 3.4. Potentiodynamic Polarization

**Figure 5:**
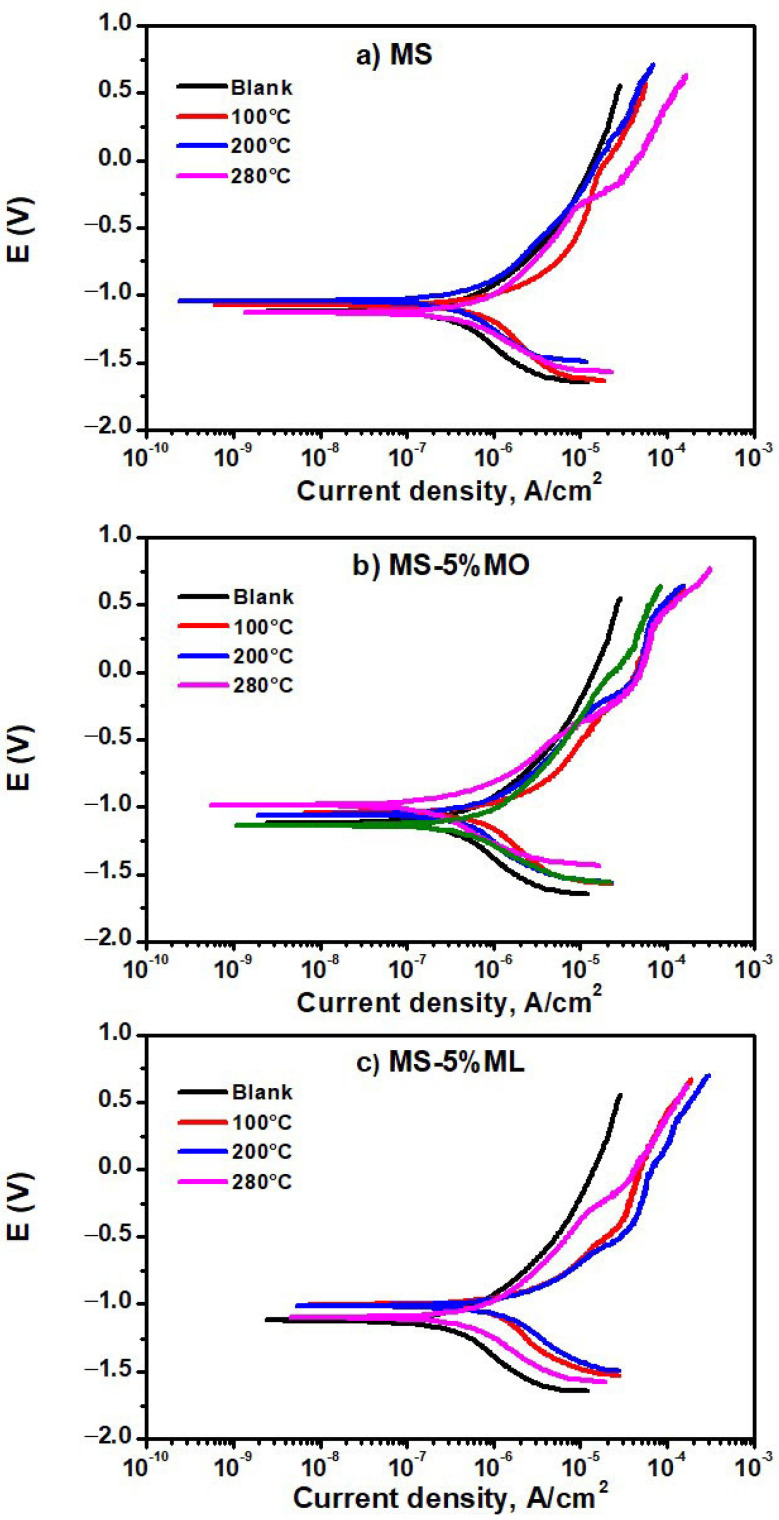
Potentiodynamic polarization curves of aqueous extract immersion media after 300 h of exposure. Liquid immersions were sampled from the (**a**) MS, (**b**) MS-5% MO, and (**c**) MS-5% ML mixtures degraded at temperatures of 100 °C (red line), 200 °C (blue line), 280 °C (pink line), and their blanks (distilled water).

Figure 5 presents the potentiodynamic polarization curves obtained from the aqueous extracts containing the thermal degradation products at 100 (red line), 200 (blue line), and 280 °C (pink line), as well as the polarization curve from distilled water (blank), of the (a) MS, (b) MS-5% MO, and (c) MS-5% ML mixtures. The polarization curve from the distilled water showed a slight variation in the corrosion potential value (−1.120 V) compared to that from the aqueous extract from the degraded MS at 100 (−1.080 V) and 200 °C (−1.050 V), but also was more active to that of the degraded MS at 280 °C. (Figure 5a). Here, the E_corr_ value is −1.130 V. From their cathodic branch, we realize that all potentiodynamic polarization curves shift to current densities more significantly than those recorded in distilled water. This shift suggests an increase in the cathodic reaction rate, given the presence of MS thermal degradation products. On the other hand, the behavior observed in the anodic branch shows similar trends in all cases. An active behavior is observed immediately above the corrosion potential, thus suggesting that the formation of an oxide layer is more notable rather than only protecting the surface of the metal. We associate this tendency with the attempt to develop a passive layer on the aluminum surface. Aluminum achieves its passivation by forming a protective film of Al_2_O_3_ and Al(OH)_3_ that limits the penetration of corrosive media [50]. The curves from the thermal degradation products at 280 °C imply that a formed pseudo-passive layer has broken. A pseudo-passive layer is an active–passive state that occurs spontaneously. As the potential increases, the corrosion products cover the metal surface thoroughly, and a passive state is reached. The disruption of the passive state was due to the interaction between the Al(OH)_3_ film and the aqueous extract. Once broken, the pseudo-passive layer attempts to regenerate by itself. Then, the pseudo-passive layer is modified by the uniform corrosion of Al, allowing for the formation of Al_2_O_3_. However, Al_2_O_3_ is unstable in water, producing Al(OH)_3_ via hydration [51].

The corresponding potentiodynamic polarization curves of the aqueous extracts for MS-5% MO (Figure 5b) and MS-5% ML (Figure 5c) mixtures suggest that aluminum affects the thermal degradation products. The curves also show that the degradation temperature of the methyl ester mixtures is relevant since the response to the polarization differs from that in distilled water. This indicates that the species produced from all the thermal degradation are trapped in the aqueous phase. A passive layer on the aluminum surface is formed, which breaks down (considering −0.2 and −0.4 V for the aqueous extract of MS-5% MO and −0.3 and −0.6 V for the aqueous extract of MS-5% ML) and is generated again. Thermal residues produced from the ester mixture might be more reactive with the metal surface, causing the formation of a protective layer. Still, simultaneously, the layer would be partially dissolved, too. The formation and dissolution of the protective layer are more evident in the aqueous extracts of the MS-5% MO mixture than in the MS-5% ML mixture. The formation and dissolution of the protective layer may indicate the effect of the C=C double bonds on the aluminum surface, observing that the greater the amount of C=C double bonds present in the mixture (MS-5% ML), the less reactive the thermally generated products with the aluminum surface. Table 2 contains the electrochemical parameters E_corr_, i_corr_, β_a_, and β_c_ obtained from Tafel slopes for the MS, MS-5% MO, and MS-5% ML mixtures degraded at the temperatures chosen for this work. The current density β_a_ and β_c_ values decrease as the degradation temperature increases. Also, we observe that the corrosion rates obtained by mass loss (Figure 1) and the results from Tafel extrapolation (Table 2) seem uncorrelated. Such uncorrelation may be due to the duration of the experiments and the dissolution capacity of the thermal degradation products in the aqueous extract. We have already suggested that corrosive gaseous compounds could have been produced during the mass loss assays and interacted with the metal surface to induce mass loss.

This is relevant for MS-5% ML, which could have produced the most significant amount of corrosive and volatile compounds immediately released to the gas stream; therefore, assays at 200 and 280 °C resulted in estimations of low corrosion rates. On the other hand, the tendency of i_corr_ values reflects that the lower the temperature, the higher the corrosion rate. This behavior may result because methyl esters require more than 300 h to generate the gaseous compounds that deteriorate the metal surface. In addition, we can assume that at higher temperatures, the methyl esters potentially form insoluble oligomers in the aqueous extract; consequently, the corrosion rate is lower at higher temperatures.

### 3.5. Linear Polarization Resistance (LPR) Measurements

**Figure 6:**
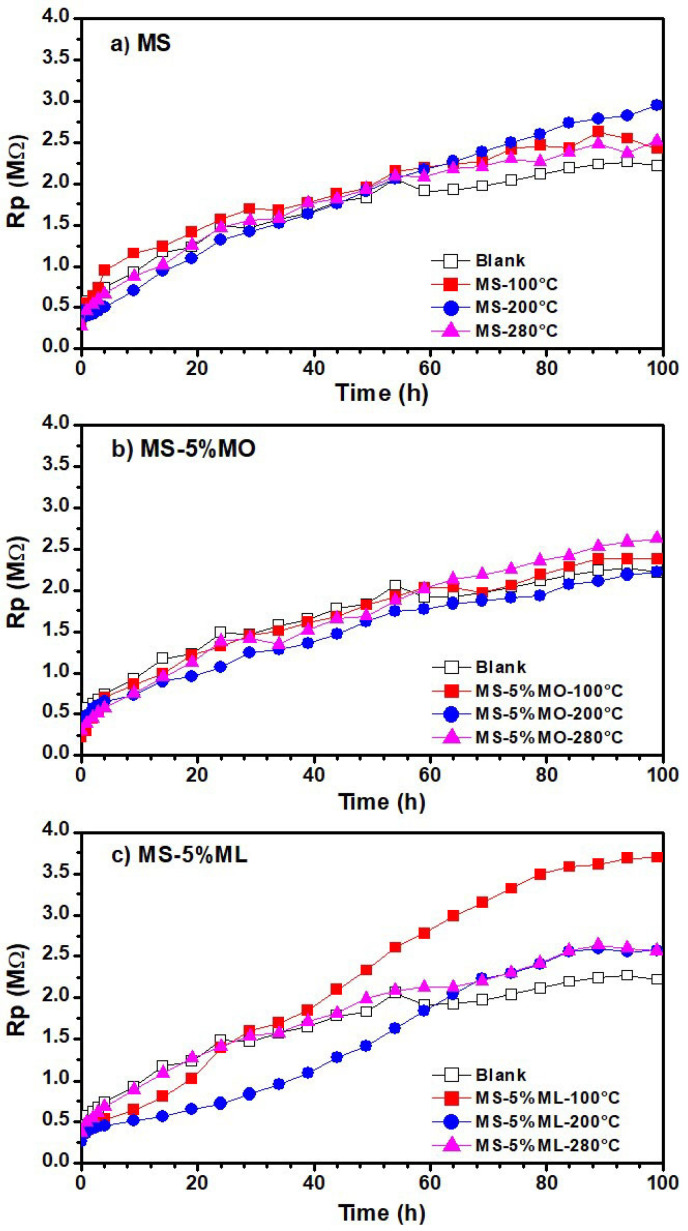
Linear polarization resistance (RPL) plots of aqueous extracts obtained from methyl ester mixtures after 300 h of degradation. Methyl ester mixtures were (**a**) MS, (**b**) MS-5% MO, and (**c**) MS-5% ML mixtures degraded at 100 °C (red line), 200 °C (blue line), 280 °C (pink line), and their blanks (distilled water).

**Figure 7:**
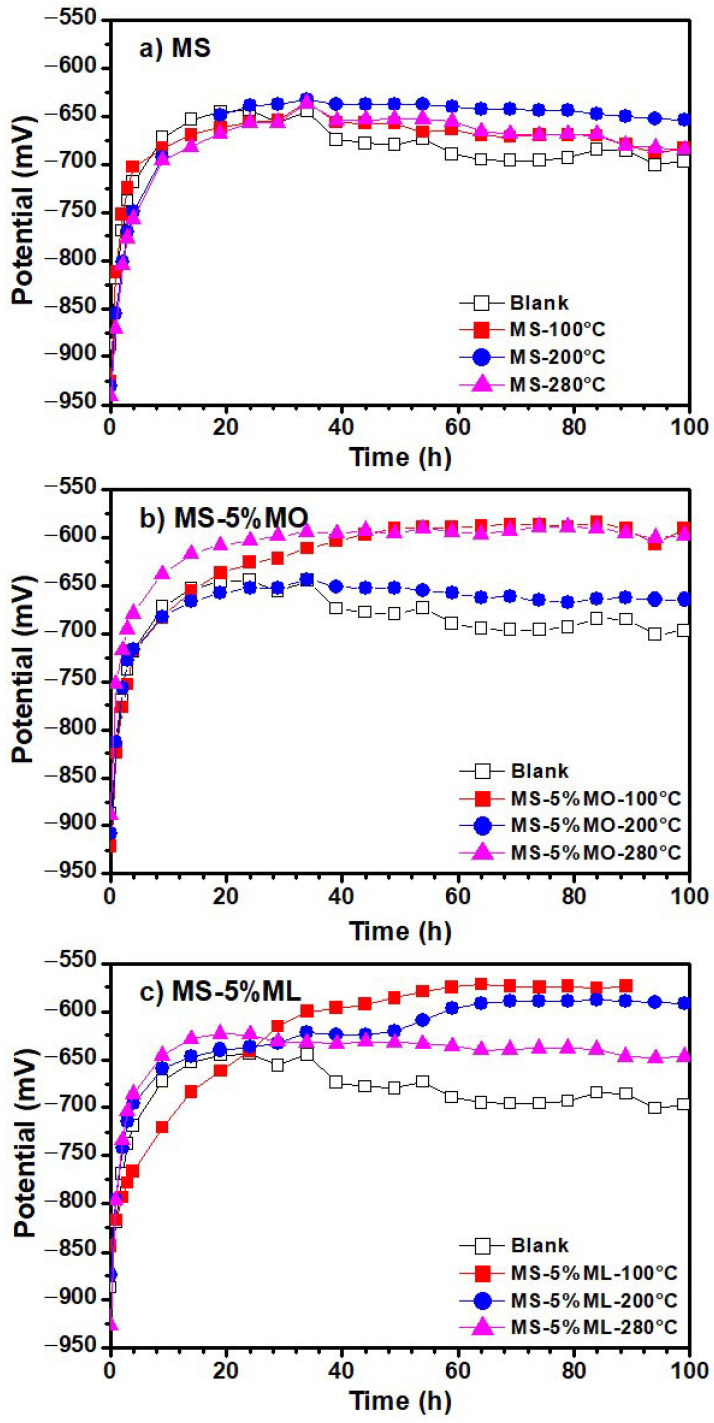
Variation in open circuit (OCP) plots of aqueous extracts obtained from methyl ester mixtures after 300 h of degradation. Methyl ester mixtures were (**a**) MS, (**b**) MS-5% MO, and (**c**) MS-5% ML mixtures degraded at 100 °C (red line), 200 °C (blue line), 280 °C (pink line), and their blanks (distilled water).

Figure 6 presents the variations of linear polarization resistance values obtained from aqueous extract immersion media after 100 h of measurements. The aqueous extracts were prepared from the (a) MS, (b) MS-5% MO, and (c) MS-5% ML mixtures degraded at temperatures of 100 °C (red line), 200 °C (blue line), and 300 °C (pink line), and a blank (distilled water). In all cases, the polarization resistance (R_p_) increases with time, indicating the formation of a passive layer on the metal surface [52]. For the MS-5% ML mixture, the R_p_ values tend to decrease with increasing temperature, suggesting that at higher degradation temperatures, the species with high interaction with the aluminum surface are formed, thus producing a passive layer. As observed in the polarization curves (Figure 5), the passive layer tends to be diluted and created again, causing fluctuation in the R_p_ values. As shown in Figure 7, which presents the variation in open circuit (OCP) plots of aqueous extract immersion media, the E_corr_ value for aluminum gradually changes positively over time with a tendency to more negative values as the temperature increases, except at 280 °C for the MS-5% MO (Figure 7b). This result is consistent with those obtained in the polarization curves, observing that the potential is slightly more positive. As reported previously, a change in E_corr_ to more positive values indicates a decrease in the corrosion rate, thus providing evidence of formation and an increase in the thickness of the passive layer [53]. These results are consistent with those obtained in potentiodynamic polarization measurements. In the OCP plots of aqueous extracts of MS-5% MO and MS-5% ML mixtures (Figure 7c), the E_corr_ values are more significant (less negative) concerning those obtained from the aqueous extract of pure MS (Figure 7a), which may indicate that the corrosion probability and the corrosion rate on the aluminum surface gradually decrease as the presence of C=C bonds increases.

### 3.6. Electrochemical Impedance Spectroscopy (EIS)

**Figure 8:**
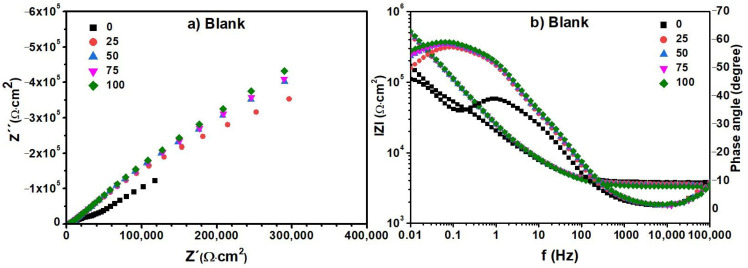
Electrochemical impedance spectra. (**a**) Nyquist and (**b**) Bode diagrams of distilled water (blank) recorded for a duration of the test of 0 (black symbol), 25 (orange symbol), 50 (blue symbol), 75 (pink symbol), and 100 h.

**Figure 9:**
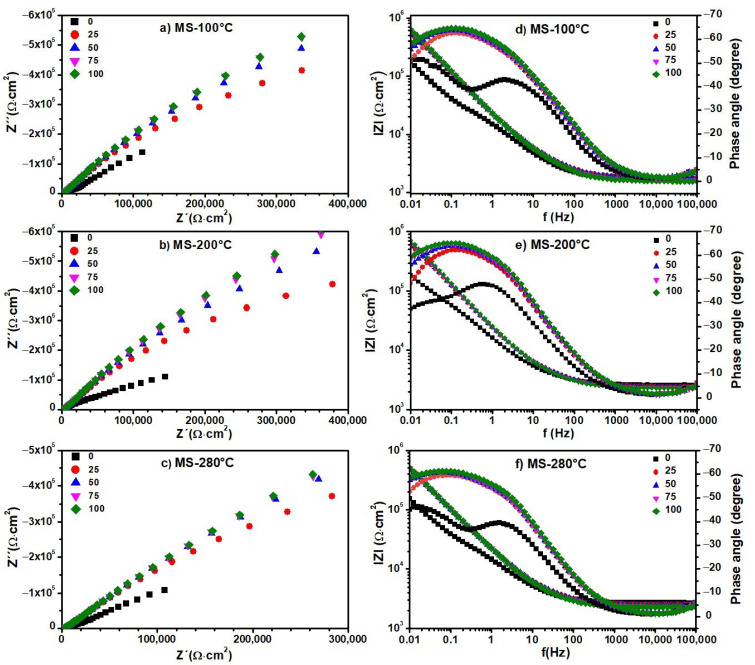
Electrochemical impedance spectra. (**a**–**c**) Nyquist and (**d**–**f**) Bode diagrams of aqueous extracts obtained from Methyl Stearate after 300 h of degradation at 100, 200, and 280 °C. Nyquist and Bode diagrams were recorded for a duration of the test of 0 (black symbol), 25 (orange symbol), 50 (blue symbol), 75 (pink symbol), and 100 h.

**Figure 10:**
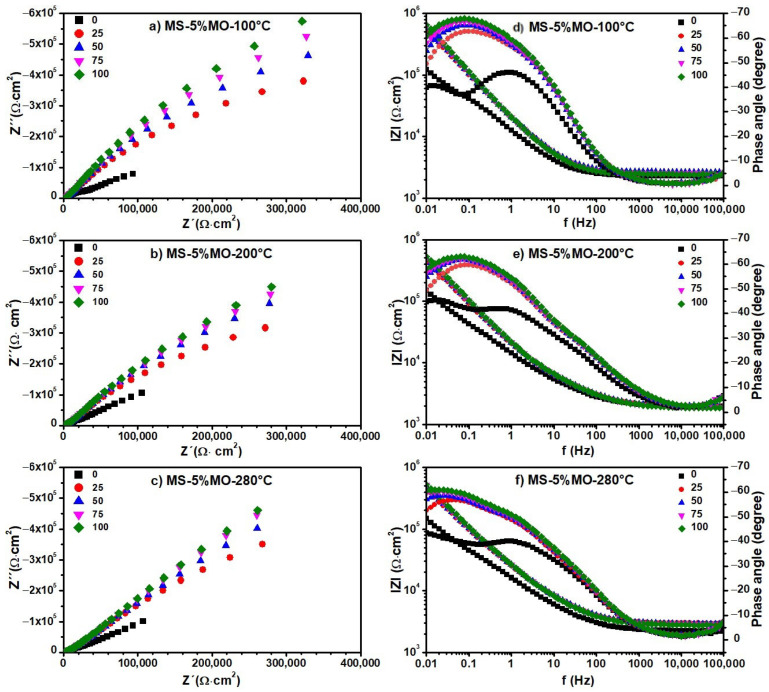
Electrochemical impedance spectra. (**a**–**c**) Nyquist and (**d**–**f**) Bode diagrams of aqueous extracts obtained from a mixture of 5% Methyl Oleate in Methyl Stearate (MS-5% MO) after 300 h of degradation at 100, 200, and 280 °C. Nyquist and Bode diagrams were recorded for a duration of the test of 0 (black symbol), 25 (orange symbol), 50 (blue symbol), 75 (pink symbol), and 100 h.

**Figure 11:**
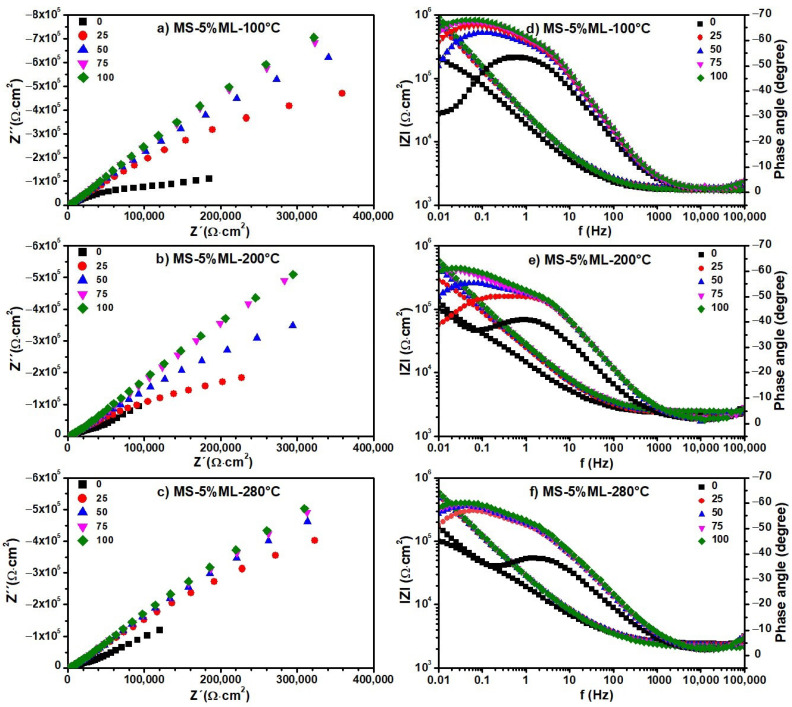
Electrochemical impedance spectra. (**a**–**c**) Nyquist and (**d**–**f**) Bode diagrams of aqueous extracts obtained from a mixture of 5% Methyl Linoleate in Methyl Stearate (MS-5% ML) after 300 h of degradation at 100, 200, and 280 °C. Nyquist and Bode diagrams were recorded for test durations of 0 (black symbol), 25 (orange symbol), 50 (blue symbol), 75 (pink symbol), and 100 h.

The Nyquist and Bode diagrams obtained from the EIS experiments on the blank, i.e., distilled water, are shown in Figure 8. The same diagrams for the aqueous extracts containing the degradation products generated at 100, 200, and 280 °C from the MS (Figure 9), MS-5% MO (Figure 10), and MS-5% ML (Figure 11) mixtures are presented for test durations of 0 (black symbol), 25 (orange symbol), 50 (blue symbol), 75 (pink symbol), and 100 h. In all cases (blank, MS, MS-5% MO, and MS-5% ML), the Nyquist spectra show a depressed semicircle centered on the axial axis. This phenomenon is called the “dispersion effect”, which appears due to the roughness, resistance to mass transport, and other heterogeneities present on the metal surface [17,28], thus causing a change in the typical response of a semicircle [54]. The semicircle is attributed to the aluminum oxide layer and the charge transfer resistance of the uniform corrosion. The shape of the semicircle remains unchanged with the lapse of time (0 to 100 h), the degradation temperature (100, 200, and 280 °C), and the type of methyl ester mixture (MS, MS-5% MO, and MS-5% ML). This may suggest that the corrosion mechanism of aluminum is carried out by charge transfer and is unaffected by exposure time, temperature, or the different aqueous extracts. Since the diameter of the semicircle extrapolated on the Nyquist diagram represents the charge transfer resistance (R_ct_), equivalent to the polarization resistance (R_p_), the trend observed in the Nyquist diagrams is consistent with the values obtained from LPR. In all cases, an increase in the diameter of the semicircle is observed over time, indicating an increase in R_ct_ and, consequently, a decrease in the corrosion rate. The Bode diagram lets us know about the surface processes occurring in the work metal. A previous report [30] bases the interpretation on frequency intervals, where the high-frequency region is associated with the resistance of the electrolyte. The low-frequency region is often related to surface processes such as charge transfer or other processes on the metal–electrolyte interface (adsorption). In the high-frequency region, the Bode plots show a straight horizontal line in the different aqueous extracts of the degraded esters, showing evidence of high resistance to the solution, observing that the resistance to the solution is directly proportional to the degradation temperature.

In the high-frequency region, the impedance modulus (lZl) value decreases with time, while the phase angle (θ) changes from 0 to −5. At intermediate frequencies, we realize that the log lZl and the log f have a linear relationship whose slope is -1 for times later than the initial one. This result can reveal the effect of the C=C double bond in the esters on the degradation products, indicating that potentially, the greater the amount of C=C bonds, the more significant the polarization resistance is. In the low-frequency region, lZl presents high values, showing that the impedance is directly proportional to the exposure time in the ester mixture at different degradation temperatures. High impedance values in this frequency region have been associated with the passive film barrier layer [55]. This indicates that the resistance to polarization increases with exposure time in the medium. In turn, the value of θ also increases with time. However, the lZl value decreases when the metal is in contact with the products generated at temperatures 200 and 280 °C. For longer onset times, the phase angle plots show a single peak at low frequencies with values between 60 and 70°.

Also, for the initial time, two peaks are formed, one at frequencies from 1 to 100 Hz and another below 0.1 Hz, whose maximum value is lower than those shown at later times (−40, −45°). These peaks indicate the formation and evolution of one and two time constants. This time constant can be associated with charge transfer processes on the metal surface. The phase angle peak becomes broad with time-lapse, confirming a low corrosion rate. At the end of the test (100 h), it was found that the value of θ decreases with the increasing degradation temperature of the esters, showing that the aqueous extract of the MS-5% ML mixture has the smallest values.

**Figure 12:**
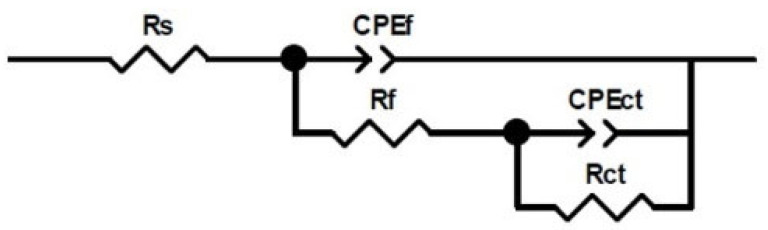
Proposed equivalent circuit for the metal–aqueous extract interface at different immersion times.

The impedance results can be interpreted using an equivalent circuit shown in Figure 12, from which the parameters of interest were obtained. According to previous reports [56], aluminum’s electrochemical behavior is determined by the presence of two layers: the outer porous layer and the inner barrier layer.

The inner barrier layer is compact and remains in direct contact with the metal, forming instantly when the metal comes into contact with an oxidizing medium. The rate of formation is independent of the oxygen partial pressure. The second layer, the external porous layer, grows on top of the internal one through a reaction with the external environment. This second film is less compact and porous than the barrier layer [56]. In the electrical circuit, these layers are represented as R_ct_ and R_f_, which correspond to the resistance of the inner porous layer and the resistance of the outer barrier layer, respectively.

R_s_ corresponds to the resistance associated with the electrolyte, while the constant phase element (CPE) represents the surface’s heterogeneity. The impedance of the CPE can be described using Equation (2),

(2) $$Zcpe=[{Q\left({jw)}^{n}\right]}^{-1}$$

where *j* is the imaginary number; *Q* is the real constant independent of frequency; *ω* is the angular frequency (rads^−1^) given by ω = 2πf; f is the frequency of the applied signal; and n is the CPE exponent with values of 1, 0, −1 and indicates surface homogeneity. So, the more heterogeneous the surface, the higher the value of *n* is [57]. To calculate the effective capacitance of the double layer (*C*) from a CPE, Equation (3) is used:

(3) $$C={\left[QR^{\left(1-\alpha \right)}\right]}^{\frac{1}{\alpha }}$$

**Figure 13:**
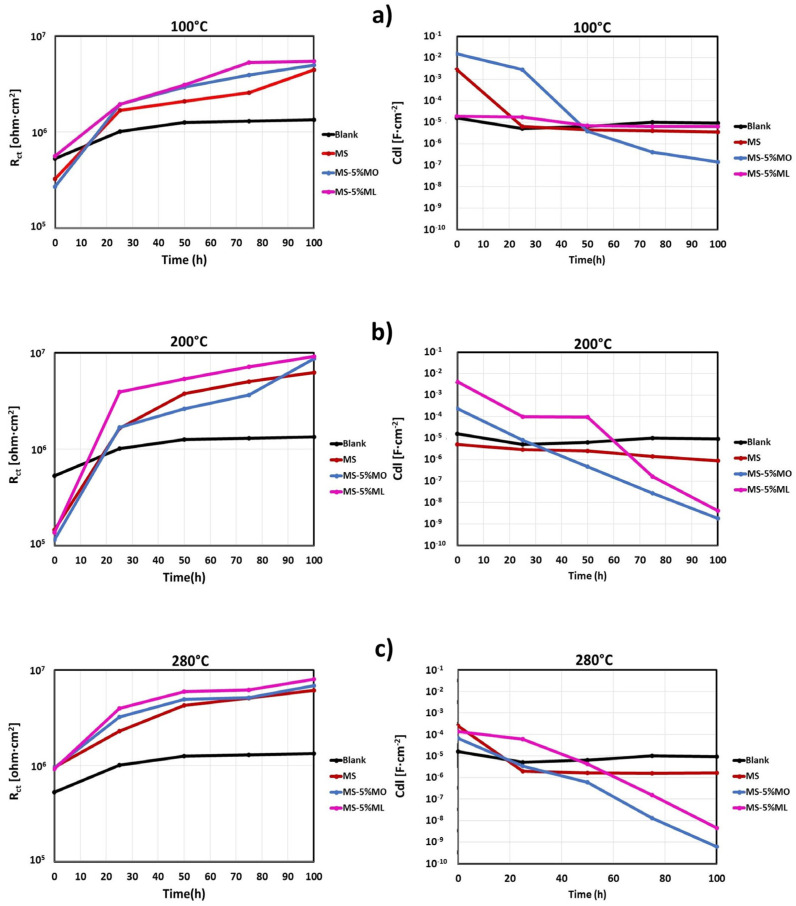
R_ct_ and Cdl values as a function of exposition time and degradation temperature for the liquid immersion media of blank (distilled water), Methyl Stearate (MS), 5% Methyl Oleate in Methyl Stearate (MS-5% MO), and 5% Methyl Linoleate in Methyl Stearate (MS-5% ML), after 300 h of the metal samples exposed. Liquid immersions were sampled at (**a**) 100, (**b**) 200, and (**c**) 280 °C.

The values of the electrochemical parameters *R_ct_* and double-layer capacitance (Cdl) obtained from fitting EIS data (Figure 8, Figure 9, Figure 10 and Figure 11) are given in the curves of Figure 13 at the degradation temperatures of (a) 100 °C, (b) 200 °C, and (c) 280 °C. Table 3 also presents the values for the electrochemical parameters extracted from the fitting processes.

We obtained such values by considering the electric circuit shown in Figure 12. EIS data under fitting processes included those obtained from the blank (distilled water) and all the aqueous extracts. For the blank, values for R_ct_ ((1.23 ± 0.15) × 10^6^ ohm·cm^2^) and Cdl ((7.69 ± 2.33) × 10^−6^ F·cm^−2^) remained closely invariant for EIS measurements above 25 h.

In contrast, the tendencies for both electrochemical parameters are different for the aqueous extracts. Here, the R_ct_ values increase while those for Cdl decrease through time in all thermal degradation conditions. An increasing tendency on the R_ct_ values suggests that the impedance on the aluminum surface with the electrolyte also increases with time. Our R_ct_ values agree with the polarization resistance (Rp) tendencies shown previously (See Section 3.5), thus confirming the formation of a passive layer on the aluminum surface [52] according to the time. However, some significant differences must be pointed out even when tendencies on the Rct and Cdl values throughout the time are similar for the many aqueous extracts. As the degradation temperature increases, R_ct_ values increase; the largest ones are those at 200 °C in each mixture; see Table 3. Also, R_ct_ values show an increasing tendency among the aqueous extracts obtained from the methyl ester mixtures, from MS to the MS-5% ML. Such a tendency reveals that the C=C bond must affect the impedance behavior due to the chemical species contained in the extracts. Cdl fittings also reveal an effect of the C=C bond, considering that the smallest values correspond to the MS-5% MO and MS-5% ML mixtures as the degradation temperature increases. The decrease in the Cdl parameter is associated with lower dielectric constants and thicker electric double layers. Thus, due to the degradation temperatures and the unsaturation C=C in methyl esters, chemical species in the aqueous extracts should have interacted more with the aluminum surface, implying that the passive layer may be more compactly arranged [58].

## 4. Conclusions

Using electrochemical techniques allowed us to investigate aluminum corrosion in aqueous extracts containing products from the degradation of methyl ester mixtures (pure MS, MS-5% MO, and MS-5% ML) at different temperatures (100, 200, and 280 °C) in free-oxygen environments. Our potentiodynamic polarization studies revealed that the aqueous extract of the mixture MS-5% ML resulted more aggressively with the aluminum surface, compared to MS or MS-5% MO, at the corresponding exposure temperatures. The polarization resistance (R_p_) values suggest the formation of a passive layer on the metal surface for all aqueous extracts of the methyl ester mixtures. These values tend to decrease with increasing temperature for the MS-5% ML mixture, suggesting that at higher degradation temperatures, the thermally degraded species of this mixture have a high interaction with the aluminum surface, producing an effective passive layer. The impedance assays revealed that all aqueous extracts of the media’s corrosion processes studied are similar (generating an arc) and remain unaltered with exposure time. R_ct_ and Cdl values obtained from EIS data agree with the RPL results and provide additional support in forming a passive layer according to the degradation temperature and the type of aqueous extract. However, we also observed that unsaturation in the degraded esters generates lZl and phase angle values similar to those of MS extracts. Consequently, the double bond’s influence on the active–passive state of aluminum still requires additional investigation. Finally, the loss mass assays allowed us to estimate high corrosion rates on aluminum exposed to thermally degraded products of MS, which potentially had enough time to interact with the metal surface. The mixtures containing MO or ML induced lower corrosion rates due to thermal product volatility.

## Figures and Tables

**Table 1:** Main products detected in the methyl ester mixtures (MS, MS-5% MO, and MS-5% ML) degraded at 200 and 280 °C via GC/MS analysis.

Methyl Stearate (MS)				
Temperature	No.	Product	No.	Product
200	1	Eicosanoic acid, methyl ester	3	Hexadecanoic acid, methyl ester
	2	Octadecanoic acid, methyl ester		
280	2	Octadecanoic acid, methyl ester	5	Decanedioic acid, dibutyl ester
	4	Heptadecanoic acid, methyl ester	6	n-Heptadecane
5% Methyl Oleate in Methyl Stearate (MS-5% MO)				
	1	Eicosanoic acid, methyl ester	8	Octadecanoic acid, octyl ester
200	7	Nonadecanoic acid, methyl ester	4	Heptadecanoic acid, methyl ester
	2	Octadecanoic acid, methyl ester	3	Hexadecanoic acid, methyl ester
280	9	n-Hexatriacontane	13	n-Nonadecane
	10	n-Pentatriacontane	14	n-Octadecane
	11	n-Heneicosane	15	Oxiraneoctanoic acid, 9-oxo methyl ester
	12	n-Eicosane		
5%Methyl Linoleate in Methyl Stearate (MS-5% ML)				
200	1	Eicosanoic acid, methyl ester	3	Hexadecanoic acid, methyl ester
	2	Octadecanoic acid, methyl ester	6	n-Heptadecane
	8	Octadecanoic acid, octyl ester	16	Bis(2-ethylhexyl)phthalate
280	10	n-Pentatriacontane	18	n-Pentadecane
	14	n-Octadecane	2	Octadecanoic acid, methyl ester
	6	n-Heptadecane	5	Decanedioic, dibutyl ester
	17	n-Hexadecane		

**Table 2:** Electrochemical parameters extracted from the potentiodynamic polarization curves in Figure 5.

Methyl Ester Mixture	T (°C)	E_corr_ (V)	i_corr_ (µA/cm^2^)	β_a_ (mV/Dec)	β_c_ (mV/Dec)
Blank		−1.120	0.529	443.9	737.1
MS	100	−1.080	0.913	304.2	738.9
	200	−1.050	0.566	421.4	624.7
	280	−1.130	0.472	285.3	397.5
MS-5% MO	100	−1.050	0.895	279.8	705.9
	200	−1.060	0.476	270.9	524.1
	280	−0.989	0.185	196.7	376.4
MS-5% ML	100	−1.003	1.260	273.5	695.8
	200	−1.010	1.370	272.0	523.6
	280	−1.100	0.544	292.0	457.7

**Table 3:** Electrochemical parameter (R_ct_ and Cdl) values. Such values were obtained from the fitting procedure over the electrochemical impedance spectroscopy curves (Figure 8, Figure 9, Figure 10 and Figure 11), considering the proposed equivalent circuit for the metal–aqueous extract interface in Figure 12.

Methyl Ester Mixture		100 (°C)		200 (°C)		280 (°C)	
	Time (h)	R_ct_ × 10^6^ (Ω·cm^2^)	Cdl × 10^−6^ (F·cm^2^)	R_ct_ × 10^6^ (Ω·cm^2^)	Cdl × 10^−6^ (F·cm^2^)	R_ct_ × 10^6^ (Ω·cm^2^)	Cdl × 10^−6^ (F·cm^2^)
Blank	0	0.526	16.00				
	25	1.010	5.110				
	50	1.260	6.370				
	75	1.290	9.970				
	100	1.340	9.320				
MS	0	0.325	2890	0.145	5.010	0.959	245
	25	1.680	6.260	1.640	2.980	2.290	2.010
	50	2.090	4.350	3.760	2.510	4.270	1.670
	75	2.550	4.140	5.000	1.390	5.090	1.600
	100	4.430	3.520	6.250	0.883	6.090	1.620
MS-5% MO	0	0.271	15,600	0.115	240	0.951	67.20
	25	1.950	2800	1.690	8.220	3.210	3.35
	50	2.940	3.810	2.630	0.471	4.890	0.588
	75	3.920	0.418	3.660	0.027	5.140	0.0127
	100	4.950	0.143	8.680	0.002	6.860	0.001
MS-5% ML	0	0.561	19.400	0.137	4220	0.920	136.00
	25	1.940	17.300	3.920	101.000	3.960	60.000
	50	3.082	7.050	5.320	94.500	5.950	4.350
	75	5.312	6.510	7.110	0.165	6.190	0.154
	100	5.442	6.500	9.090	0.004	7.980	0.005

## Data Availability

Data are contained within the article.
